# Recombinant soluble gp130 protein reduces DEN-induced primary hepatocellular carcinoma in mice

**DOI:** 10.1038/srep24397

**Published:** 2016-04-15

**Authors:** Jing Hong, Hang Wang, Guoying Shen, Da Lin, Yanxue Lin, Nanhui Ye, Yashan Guo, Qiaoling Li, Nanhui Ye, Chengjun Deng, Chun Meng

**Affiliations:** 1 Institute of Pharmaceutical Biotechnology and Engineering, College of Biological Science and Biotechnology, Fuzhou University, Fuzhou, Fujian, 350002, China

## Abstract

IL-6 (interleukin 6) plays an important role in the development and growth of hepatocellular carcinoma (HCC) via both classic signaling and trans-signaling pathways. Soluble gp130 (sgp130) is known to be a natural inhibitor of the trans-signaling pathway. In the present study, our goal was to investigate whether recombinant sgp130 could suppress the initiation and progression of HCC in mouse models. Our results demonstrate that sgp130 induced an apoptosis of HepG2 cells and inhibited the clonogenicity of HepG2 *in vitro*. Moreover, the IL-6 trans-signaling pathway is significantly suppressed by sgp130 as reflected by the decrease in the level of STAT3 phosphorylation and other inflammatory factors both *in vitro* and *in vivo*. In the DEN-induced HCC mouse model, intravenous injection of sgp130 attenuated hepatic fibrosis at 16 weeks and reduced the initiation and progression of primary HCC at 36 weeks. Furthermore, our results also demonstrate that intravenous administration of sgp130 significantly suppressed the growth and metastasis of xenograft human HCC in NOD/SCID mice.

Worldwide in 2012, the five most common cancers among men included lung, prostate, colorectal, stomach and liver cancers^1^. In 2010, as many as 418,000 new cases of liver cancer were diagnosed in China alone and out of these, 380,000 patients died due to this disease. As a result, liver cancer is regarded as one of the most lethal of all cancers and unfortunately there is no effective drug or means available for the treatment or cure of this deadly disease.

Hepatocellular carcinoma (HCC), which accounts for 80% of primary liver cancers, is associated chiefly with inflammation-damaged livers resulting from viral hepatitis infections (hepatitis B or C), or from cirrhosis due to chronic alcoholism^2^. IL-6 is one of the key cytokines mediating both the inflammatory response and HCC development in liver^3^,^4^,^5^,^6^,^7^,^8^. Furthermore, IL-6 modulates the expression of several liver cancer specific genes during the inflammatory state, and autocrine IL-6 is crucial for the initiation and progression of HCC^9^. The elevated levels of IL-6 frequently detected in cancer patients correlate with a poor prognosis in these patients.

IL-6 signals via two distinct pathways as IL-6 receptors exist in both membrane-bound (mIL-6R) and soluble (sIL-6R) forms. Both pathways require a dimer of membrane-bound gp130 molecules for the signal transduction. IL-6 binds mIL-6R in the classic signaling pathway while it binds soluble IL-6R in the trans-signaling pathway. The classic signaling pathway mainly mediates anti-inflammatory effects while the trans-signaling pathway mediates pro-inflammatory effects and cancer development. Numerous experimental and clinical findings have indicated that IL-6 and the partners in its signaling pathways are valid drug targets for many different types of cancers^10^.

Recent studies have demonstrated that targeting the trans-signaling pathway may be an efficient avenue for anti-HCC therapeutics^11^,^12^,^13^. Soluble gp130 (sgp130) is known to be a natural inhibitor of the trans-signaling pathway. A modified sgp130 linked to an Fc fragment has been reported to have significant efficacy both for reducing pancreatic cancer growth and also for preventing tumor recurrence in orthotropic xenograft mouse models^14^. In the present study, our goal was to investigate whether recombinant sgp130 could effectively suppress the IL-6 trans-signaling pathway and thereby suppress HepG2 cell growth as well as the development of HCC in DEN-treated mice.

## Results

### Recombinant sgp130 induces apoptosis of HepG2 cells *in vitro*

**Figure 1:**
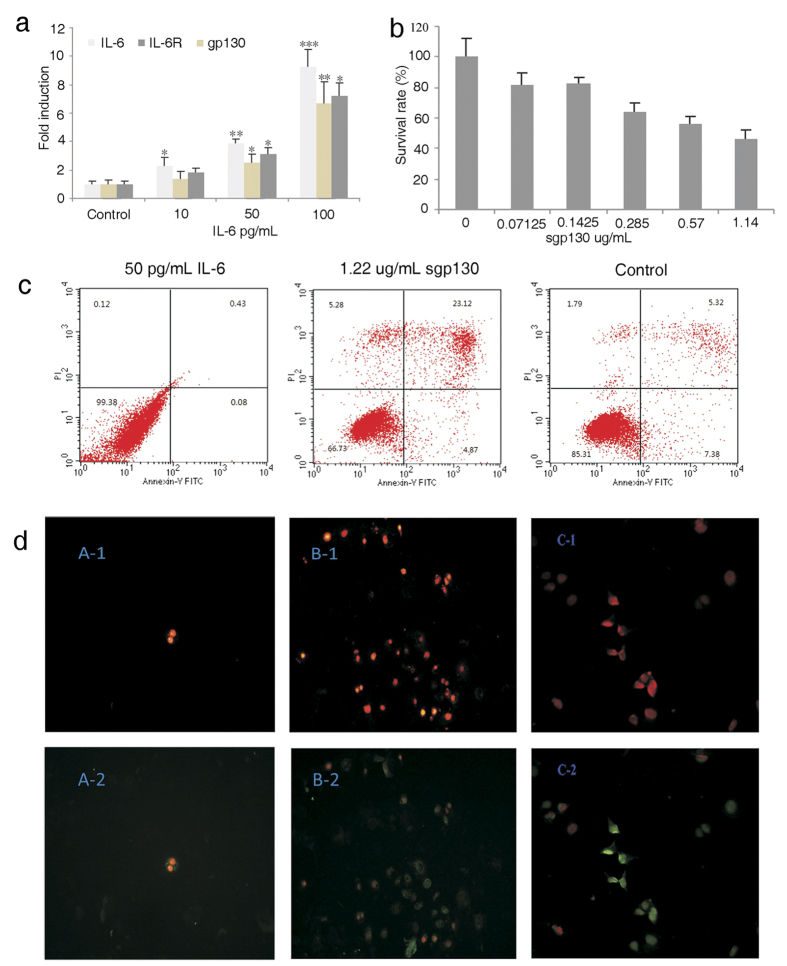
Sgp130 influences the growth and apoptosis of HepG2 cells. (**a**) IL-6 increases the transcriptional level of IL-6 signaling-related genes in a dose-dependent manner (Single*, double** and triple*** marked represent statistical significance in *p* < 0.05, *p* < 0.01 and *p* < 0.001 in comparison with their control samples, respectively). (**b**) sgp130 inhibits HepG2 cell growth in a dose-dependent manner as measured with MTT. (**c**) Apoptosis of HepG2 cells analyzed with FACS after IL-6 or sgp130 treatment for 24 h. (**d**) Observed with the PI and Annexin-FITC stained HepG2 cells before FACS analysis (red image observed with UV laser; green image observed with blue light).

IL-6 is one of the best-characterized of the tumorigenic cytokines that promote tumor initiation, progression, and metastasis by activating inflammatory responses^3^,^15^. High levels of IL-6 expression correlate well with advanced stages and metastasis of tumors. We first investigated the relationship between added recombinant IL-6 and the IL-6 expression in HepG2 cells. We found a 2−, 4− and 8-fold increase of IL-6 transcription in HepG2 cells after adding 10, 50 and 100 pg/mL respectively of exogenous IL-6 in culture media. Interestingly, we also observed a similar dose-dependent increase of transcriptional levels of IL-6R and gp130 albeit in a somewhat lower magnitude (Fig. 1a).

Next, we sought to examine whether recombinant sgp130 alone was sufficient to inhibit the growth of HepG2 cells presumably by competing with the binding of endogenous gp130 to IL-6/IL-6R. We added a series of increasing concentrations of recombinant sgp130 protein in the culture media and then monitored the growth of HepG2 cells (Fig. 1b). The MTT cell proliferation assay revealed that sgp130 significantly inhibited the proliferation of HepG2 cells in a dose-dependent manner with an IC50 of 1.22 μg/mL. The observation of cells stained with Annexin V- FITC and PI, showed that sgp130 also induced an apoptosis of HepG2 cells. Flow cytometry analysis further showed that sgp130 induced significant apoptosis compared with control cells (5.32%) and IL-6 treated cells (0.23%) in 24 h. Addition of 1.22 μg/mL recombinant sgp130 resulted in about 23% HepG2 cell apoptosis (Fig. 1c,d).

### Recombinant sgp130 inhibits IL-6 signaling pathway

**Figure 2:**
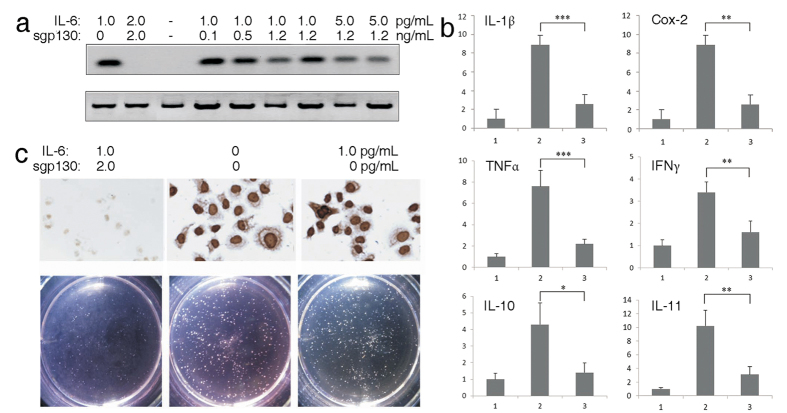
Sgp130 suppresses IL-6 signaling pathway in HepG2 cells. (**a**) Lysates of HepG2 cells treated with IL-6 and sgp130 for 48 h were analyzed for global tyrosine phosphorylation at position 705 of STAT3. (**b**) sgp130 inhibits IL-6 induced transcriptional activation of inflammatory factors in HepG2 cells treated with sgp130 or IL-6 for 48 h (1: control; 2: positive sample; 3: sgp130 treated sample). Relative expression levels of cytokine mRNAs isolated from cells and analyzed by real-time PCR. Data are mean ± SD. n = 3 (Single*, double** and triple*** marked represent statistical significance in *p* < 0.05, *p* < 0.01 and *p* < 0.001, respectively). (**c**) sgp130 decreases IL-6-induced increase of Ki67 in IL-6 and sgp130 co-treated HepG2 cells for 48 h. The cells were stained with a specific antibody against Ki67 and the treatment followed with HRP-coupled secondary antibody (upper panel); sgp130 suppresses IL-6 activated growth of HepG2 cells which was analyzed by soft agar assay (lower panel). (1 × 10^3^ cells/well).

The IL-6 signaling pathway is activated when IL-6 binds to soluble or membrane-bound IL-6 receptor (IL-6Rα), which interacts with membrane-bound gp130 subunit, and triggers downstream effectors. STAT3 is recognized as the major signal transducer downstream of the IL-6 signaling pathway and functions as an oncogene and a key factor associated with the process of inflammation and carcinogenesis. STAT3 phosphorylation is a major event on the activated IL-6 signaling pathway^16^. To assess the influence of sgp130 on the JAK/STAT mediated IL-6 signaling pathway, the phosphorylation status of tyrosine 705 in STAT3 was analyzed by Western blot in HepG2 cells. Sgp130 significantly decreased the STAT3 phosphorylation induced by IL-6 (Fig. 2a). Furthermore, the effect of the recombinant sgp130 on more extensive inflammatory responses in IL-6 treated HegG2 cells was analyzed by quantitative RT-PCR. Recombinant sgp130 significantly reduced the elevated expression of a few key inflammatory factors such as IL-1β, Cox-2, TNFα, IFNγ, IL-10 and IL-11 (Fig. 2b). The expression of Ki67, a marker of cell proliferation, was also reduced indicating that recombinant sgp130 has an inhibitory effect on HepG2 cell proliferation (Fig. 2c upper panel).

Using a clonogenicity assay, we further investigated the effect of recombinant sgp130 on HepG2 cell proliferation by comparing the results with that induced by IL-6. Recombinant sgp130 significantly inhibited colony formation of HepG2 cells, whereas IL-6 significantly promoted cell proliferation and induced bigger and greater number of cell colonies than the non-treated controls (Fig. 2c lower panel). These results further indicate that recombinant sgp130 is capable of inhibiting tumor growth.

### Recombinant sgp130 decreases inflammatory response in DEN-induced mouse livers

**Figure 3:**
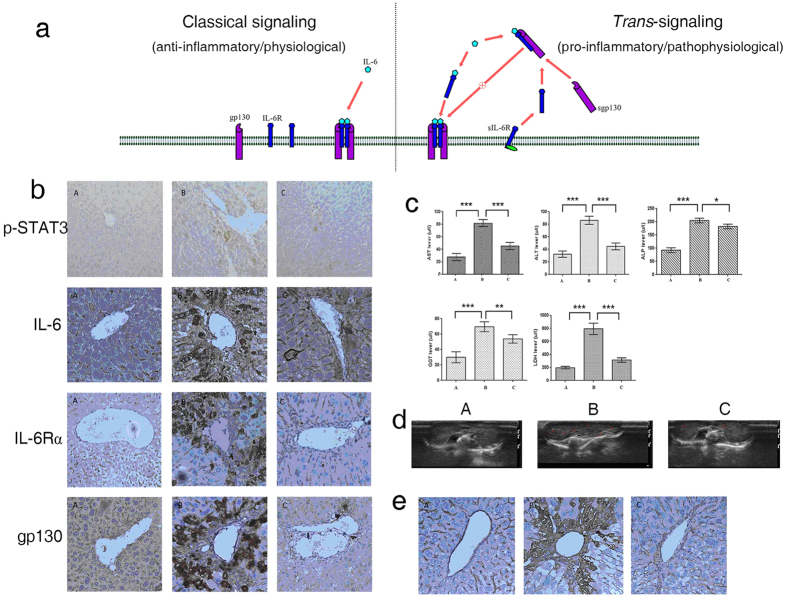
Sgp130 reduces DEN-induced inflammatory responses in mouse liver tissue at 16 weeks. (**a**) Schematic diagrams of sgp130 in inhibiting IL-6 signaling. (**b**) Expression of p-STAT3, IL-6, IL-6R and gp130 in mouse liver tissue (400×). (A: Control; B: model group; C: sgp130 treatment group). (**c**) sgp130 changes the DEN-induced high level of liver related factors in mouse sera. (**d**) B-ultrasonography of liver for detecting DEN-induced hepatic fibrosis (Arrowheads point to hepatic fibrosis bright spots or stripes; (A: Control; B: model group; C: sgp130 treatment group)). (**e**) type IV collagen expressed in tissue of mouse livers, detected with immunohistochemical staining (400×) (A: Control; B: model group; C: sgp130 treatment group).

Diethyl nitrosamine (DEN) significantly increases the expression of IL-6, IL-6R and gp130, the main partners of IL-6 pathway, and induces serious inflammatory reactions in the mouse liver. In contrast, recombinant sgp130 binds to IL-6/sIL-6R complex in competition with membrane-bound gp130 to inhibit the activation of IL-6 signaling pathway as proposed in the schematic diagram in Fig. 3a^10^.

To investigate the effect of sgp130 on inflammation and cancer development in mouse liver, high doses of DEN/CCl_4_ (100 mg/kg i.p.) were used to treat 15-day-old BLBA/C mice, which were subjected to intravenous injections of recombinant sgp130. The phosphorylation of STAT3 was evaluated to confirm the activation of IL-6 pathway *in vivo*. STAT3 phosphorylation was significantly increased in the livers of DEN-treated mice while a similar increase was not observed in the livers of non-treated control mice. Administration of sgp130 decreased the phosphorylation of STAT3 (Fig. 3b) and also decreased the immunostaining of IL-6, IL-6R and gp130 in mouse livers induced by DEN treatment. These data indicate that recombinant sgp130 can inhibit the inflammation reactions in the livers of DEN-treated mice and can thereby protect the liver tissues from inflammatory injury *in vivo*.

To further investigate whether the elevated STAT3 phosphorylation correlates well with the liver tissue damage caused by the DEN-induced inflammation, we analyzed a few established liver specific biomarkers such as alanine aminotransferase (ALT) and aspartate aminotransferase (AST) in the mouse bloodstream (Fig. 3c). High levels of ALT and AST were detected in the bloodstream of DEN-treated mice. Administration of recombinant sgp130 effectively reduced these elevated biomarkers. Other biomarkers in bloodstream, though less specific to liver tissue damage, such as alkaline phosphatase (ALP), gamma-glutamyl transferase (GGT) and lactate dehydrogenase (LDH), are also commonly associated with liver tissue damage caused by hepatitis or HCC and were also seen to be markedly elevated in the blood of DEN-treated mice. The elevations of ALP, GGT and LDH were all significantly reduced by the treatment with recombinant sgp130. Taken together, these results further strengthen the notion that recombinant sgp130 could specifically block the activated IL-6 signaling pathway and thereby protect the liver cells from inflammatory damage induced by DEN *in vivo*.

Ultrasound is one of the most commonly used non-invasive clinical methods for liver examination and is widely utilized for assessing liver fibrosis formation in human as well as in animals. For instance, Ho *et al.* have previously assessed liver fibrosis in DEN-treated mice by detecting the formation of fibrotic structures, that are seen as increased scatters of intense echo signals (bright dots or stripes), using B mode ultrasonography^17^. Our B mode ultrasonography results showed significant liver fibrosis at 16 weeks after DEN treatment, while administration of recombinant sgp130 largely weakened these ultrasonography signals of fibrosis (Fig. 3d).

Type IV collagen staining is another strong indicator of liver fibrosis formation and very low level of type IV collagen can be detected in liver tissue under normal physiological conditions^18^,^19^,^20^,^21^,^22^. A heavy accumulation of type IV collagen in the livers of DEN-treated mice was observed in our experiments using immunohistochemical staining. Administration of recombinant sgp130 significantly decreased the accumulation of type IV collagen in the livers of DEN- treated mice (Fig. 3e). These results further demonstrated that recombinant sgp130 reduces the degree of liver fibrosis through its anti-inflammatory effects.

### Recombinant sgp130 inhibits DEN-induced HCC development in mouse liver

**Figure 4:**
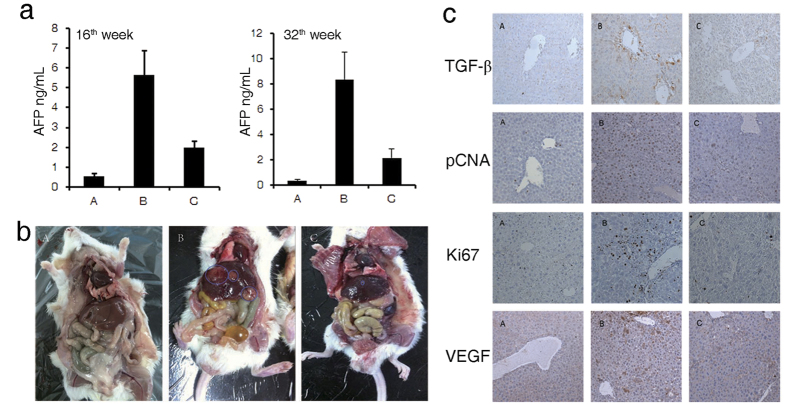
Sgp130 effects tumor initiation and development in mouse liver. (**a**) The changes of α-AFP levels in mouse sera detected at 16 weeks and 36 weeks respectively. (**b**) Expression of liver cancer marker genes in mouse liver tissue analyzed with immunohistochemical staining at Week 16 (100×). (**c**) Tumors on the surface of mouse livers after treatment for 36 weeks. (A: Control; B: model group; C: sgp130 treatment group).

To investigate whether recombinant sgp130 has any therapeutic effects on the development of HCC induced by DEN, we injected sgp130 intravenously into DEN-treated mice. We used Alpha-fetoprotein (AFP), a well-established HCC biomarker, to detect and monitor the development of HCC in DEN-treated mice. Serum AFP increased from 0.36 ng/mL in the beginning to 5.66 ng/mL at 16 weeks in the DEN-treated mice. AFP level in recombinant sgp130-treated mice was reduced to 1.98 ng/mL at 16 weeks, while at 36 weeks the AFP level decreased to 2.17 ng/mL in sgp130 treated mice versus 8.30 ng/mL in DEN-induced mice not treated with sgp130 (Fig. 4a).

Other researchers have reported a close correlation between enhanced STAT3 and transforming growth factor-β (TGFβ) signaling during the development of HCC. Therefore, we sought to examine whether TGFβ was activated in the livers of DEN-treated mice. Our immunohistochemical analysis revealed that TGFβ expression was significantly increased 36 weeks after DEN treatment in comparison with the non-DEN-treated control mice while treatment with sgp130 effectively reduced the expression of TGFβ in mouse livers (Fig. 4b).

Antigen Ki67 and proliferating cell nuclear antigen (PCNA) are nuclear proteins that are associated with and necessary for cell proliferation, and are established biomarkers for cancer diagnosis. Moreover, the EGFR expression has been reported to correlate with the development of HCC^23^,^24^. Our immunohistochemical staining of liver tissues showed significant increase of Ki67, PCNA and EGFR expression in DEN-treated mice; treatment with sgp130 markedly decreased the staining of these cancer-associated biomarkers.

A single intraperitoneal (i.p.) injection of DEN into 15-day-old BL/6 mice induces HCC nodules that can be first detected at 8 to 9-months post-injection^17^. DEN-induced HCC mice can serve as a unique primary HCC model for testing experimental anti-tumor therapeutics, and may generate more relevant oncology data than xenograft models of nude or NOD/SCID mice. Tumor nodules are macroscopically evident in the livers of DEN-treated mice at 36 weeks. Our results show that the number and volume of tumor nodules at 36 weeks in the sgp130-treated mice is significantly reduced compared with that in the DEN-treated animals not treated with sgp130 (Fig. 4c). Our results indicate that recombinant sgp130 by specifically blocking the IL-6 trans-signaling pathway, might have strong effects on HCC at various stages of the tumor development and could potentially be a valuable therapeutic agent for treating HCC in humans.

**Figure 5:**
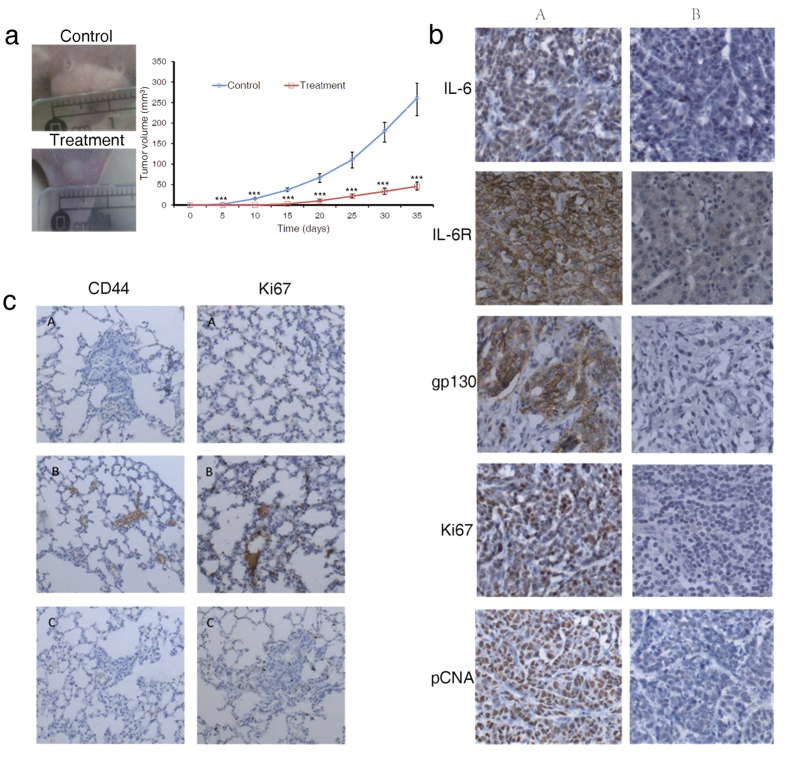
Sgp130 suppresses tumor growth in nude mice. (**a**) The xenograft tumors and their curve on nude mice receiving a subcutaneous injection of 5 × 10^6^/mL HepG2 cells. Data are mean ± SD, n = 6 (Single*, double** and triple*** marked represent statistical significance in *p* < 0.05, *p* < 0.01 and *p* < 0.001, respectively). Each experiment was repeated three times, and data shown are representative one of three independent experiments. (**b**) Immunohistochemical analysis of IL-6, IL-6R, gp130, Ki67 and PCNA expression in xenograft tumors (100×) (A: Control; B: sgp130 treatment group). (**c**) Detection of HepG2 cell metastasis in nude mouse lungs through analyzing expression of CD44 and Ki67 lung tissues (100X) (A: Negative control; B: Control; C: sgp130 treatment group).

To further confirm the therapeutic efficacy of recombinant sgp130 on HCC, we transplanted 5 × 10^6^ HepG2 cells into NOD/SCID mice in order to test the anti-tumor effects of sgp130 in a xenograft model, that could provide a convenient way for a more dynamic observation as well as a quantitative measurement of the tumor growth. Normal saline served as a negative control in the study. Our results demonstrate that recombinant sgp130 significantly reduced tumor sizes starting at Day 10 all the way to Day 35. At Day 35, the end of the study, intravenous injection of 1.14 μg/Kg sgp130 every 3 days, reduced the tumor volume 5-fold compared with the control (Fig. 5a).

Immunohistochemical analysis showed that IL-6, IL-6R and gp130 are highly expressed in tumor sections of the control NOD/SCID mice, while in sgp130-treated mice, their expression levels are significantly lower (Fig. 5b). Ki67 and PCNA, representing proliferative activities of the tumor cells, were also inhibited by sgp130 in the treated group. Observation of multiple satellite small tumors as well as positive staining of Ki67 and CD44 in the lung tissues of the control mice, but not in the lung tissues of the treated mice, indicated that tumor cells were likely to metastasize to the lungs in the control mice and also that sgp130 treatment prevented the metastases of the tumors (Fig. 5c).

## Discussion

In the present study, we demonstrate for the first time that recombinant human sgp130 significantly reduces DEN-induced primary HCC in mice and inhibits the growth and metastases of xenograft human HCC in NOD/SCID mice. Soluble gp130 (sgp130), the soluble form of membrane-bound gp130, is a naturally occurring glycoprotein that specifically inhibits soluble IL-6R mediated IL-6 trans-signaling without affecting the membrane-bound IL-6R^25^,^26^ mediated classic-signaling pathway. Considering the importance of IL-6 signaling in inflammation and cancer development, several monoclonal antibodies targeting IL-6 or IL-6R have been developed as therapeutic agents for various inflammatory diseases and cancers. Among them, toclizumab targeting the IL-6R is approved for rheumatoid arthritis, juvenile idiopathic arthritis and Castleman disease while sarilumab also targeting the IL-6R is in late stage clinical trials. However, global blockade of IL-6 signaling pathways can impact both the pro- and anti-inflammatory properties and may therefore adversely affect the immune system^27^.

IL-6 plays important roles in inflammation, immune defense, and oncogenesis, and is considered as one of the best-characterized of all the pro-tumorigenic cytokines. Activation of IL-6 signaling pathways is considered as a biomarker for various cancers^6^,^7^,^10^,^15^. Moreover, the existence of autocrine IL-6 is regarded as necessary for HCC development^9^. Recent studies have provided evidence that targeting the IL-6 trans-signaling pathway would be a valid anti-tumor approach. For instance, Hatting *et al.*, have reported that conditional deletion of gp130 in hepatocytes attenuated HCC progression in DEN-treated mice^11^. Furthermore, Goumas *et al.*, have demonstrated the efficacy of soluble gp130Fc in orthotropic xenograft models of pancreatic cancer^14^. However, there have been no studies reported thus far on the effect of either a global blockade of IL-6 signaling or a selective targeting of the trans-signaling in HCC models.

STAT3 plays a central role in the IL-6 signaling network, cross-talking with multiple signaling pathways of growth factors such as TGF, IGF, cyclin D1, D2^28^. Since soluble gp130 has been recognized as a natural inhibitor of IL-6 trans-signaling, we investigated recombinant sgp130 protein as a potential therapeutic agent targeting only the trans-signaling of IL-6 but sparing the classic pathway. As we found the presence of soluble IL-6R in HepG2 cell culture in our previous data (not shown) and observed the induction of gp130 and IL-6R by IL-6 in HepG2 cell media in the current study, it is likely that both the classic and trans-signaling pathways are intact in the HepG2 cell culture system. The induction of apoptosis and the inhibition of HepG2 cell growth, STAT3 phosphorylation, and IL-6 induced clonogenicity and inflammatory cytokines by sgp130 was most likely through the inhibition of trans-signaling pathway (shown in Figs 1 and 2).

In nearly all tumors, inflammatory pathways exist by which inflammatory cytokines and growth factors promote tumor growth and survival^29^,^30^,^31^. DEN-induced mouse HCC has been a unique paradigm to study the initiation, progression, metastasis of primary HCC and also the relationship between inflammation and tumorigenesis^9^,^11^. DEN-induced inflammation stimulates the overexpression of IL-6, IL-6R, gp130 and other pro-tumorigenic cytokines, which in turn further aggravates inflammatory damage to the liver tissues and causes cancer. In our present study, recombinant sgp130 significantly decreases STAT3 phosphorylation as well as the overexpression of IL-6, IL-6R and gp130 in DEN-treated mouse livers and strongly protects liver cells from inflammatory damage as seen by a significant decrease of liver-specific enzymes in the mouse sera. More interestingly, the reduction of fibrosis and immunostaining for type IV collagen in the sgp130 treated animals indicates that sgp130 also protects the livers from developing into cirrhosis, a property of sgp130 that can be regarded as a significant asset in addition to its anti-tumor property.

Circulating IL-6 is an important risk indicator for HCC and correlates closely with the poor prognosis of HCC patients^9^. IL-6 may also account for the lower HCC rates in females due to their lower circulating IL-6 levels than that in males. Moreover, IL-6 may promote TGFβ-dependent tumor growth and as a result, attenuation of TGFβ signaling may contribute to the reduced HCC progression in mouse livers after DEN injection^32^,^33^. We observed a significant decrease of TGFβ expression in the livers of sgp130 treated animals. Wang *et al.*^34^ have reported that the association of IL-6R and gp130 with EGFR driven by IL-6, led to EGFR-dependent re-phosphorylation of STAT3, the second wave of STAT3 activation, which further promoted IL-6-stimulated tumorigenicity by sustaining a high expression of inflammation-related proteins. The DEN-induced high expression of EGFR was significantly attenuated by sgp130 treatment in our present study. Both these above observations appear to further support our contention that a broad blockade of pathways of multiple pro-tumorigenic cytokines by sgp130 accounts for the high efficacy of sgp130 in reducing DEN-induced HCC in the mouse and also in reducing human HCC in the xenograft mouse model. The striking differences observed in the number and size of DEN-induced tumors between the sgp130-treated and control animals indicate that sgp130 may have an impact on multiple stages of HCC development such as initiation, progression as well as metastasis, as a result of the overall suppression of inflammatory reactions in the livers of sgp130-treated animals. More detailed and careful examinations in these respects are needed in future investigations. The five-fold reduction in tumor volumes, the absence of small tumors around the xenograft tumors and the absence of cancer biomarker staining in the distal organs such as lungs, observed only in the sgp130 treated NOD/SCID mice, provide further evidence that sgp130 likely inhibits both the progression and metastasis of HCC.

Both the classic and trans-signaling of IL-6 are mediated by gp130 through an identical intracellular pathway. Trans-signaling is considered a major danger signal since it enhances IL-6 responsiveness and inflammatory events^35^. Selective blockade of IL-6 trans-signaling has been proven to be more beneficial in different animal models of human diseases. For instance, Goumas and coworkers have recently demonstrated the significant efficacy of a soluble sgp130Fc, with an improved affinity for the binding of IL-6/sIL-6R, in an orthotropic xenografted pancreatic ductal adenocarcinoma (PDAC)^14^. Our present work demonstrates that a selective blockade of IL-6 trans-signaling with recombinant sgp130, in its native form without any modification, shows promising efficacy against HCC in the DEN-induced mouse model and also against xenograft human HCC in NOD/SCID mice. Thus, recombinant sgp130 may be of further interest for clinical investigation as a therapeutic drug for human hepatocellular carcinoma, which is still a challenging unmet medical need today.

## Materials and Methods

Recombinant sgp130 was generated using standard molecular cloning techniques. Briefly, the cDNA encoding sgp130 was amplified by RT-PCR from the total RNA prepared with HepG2 cells (forward and reverse primer sequences: 5′-CCCAAGCTTGCCACCATGGGGAAATATCCGCGCAAG-3′, 5′-CCCAAGCTTGCCACCATGGGGAAATATCCGCGCAAG-3′). Following digestion with restriction enzymes *Bam*HI and *Hin*dIII, the PCR fragment was cloned into pcDNA3.1 (Invitrogen, CA, USA) for eukaryotic cell expression. Recombinant sgp130 protein was generated by transfecting pcDNA3.1-sgp130 plasmid into CHO cells and purified from the collected cell medium using Ni-NTA-agarose columns (Sango Biotech) following the manufacturer’s instructions. The purified protein was further dialyzed and stored in 10% glycerol at −80 °C.

### Apoptosis analysis of HepG2 cells

The apoptosis analysis method used was described previously^36^. Briefly, HepG2 cells were harvested from 25 cm^2^ cell culture flasks at about 80% confluency and transferred to 15 ml sterile polypropylene tubes and centrifuged at 200 g for 10 min at 4 °C. The medium was removed and the cells were washed once with 4 mL of 4 °C PBS containing 1% (v/v) bovine calf serum. Each cell pellet was re-suspended in 100 μL annexin V-binding buffer containing 2 μg/mL annexin V-FITC (Caltag Laboratories, Burlingame, CA, USA) and incubated on ice for 10 min in dark. After adjusting the total volume of each tube to 0.5 mL with annexin V-binding buffer, 1 μg of propidium iodide (Sigma) was added into each tube. Cell apoptosis was analyzed with a fluorescence microscope (Eclipse TS100, Nikon, Japan) or by flow cytometry (Coulter EpicsXL, Bechman Coulter, USA), respectively.

### Cell proliferation assay

Cell proliferation assay was performed according to the conventional method. Briefly, HepG2 cell proliferation was measured by the MTT [3-(4,5-dimethylthiazol-2-yl)-2,5-diphenyl tetrazolium bromide] method. 1–2 × 10^4^ cells were plated per well in a 96-well plate in DMEM containing 10% bovine calf serum and different concentrations of sgp130 and replaced with fresh medium every 24 hrs. After 72 hrs, the medium was removed and 0.3 mL of 0.1% MTT in PBS was added into each well. Supernatant was removed and 0.8 mL of 2-propanol was added after incubating for 30 min at 37 °C. Absorption at 560 nm was measured with a plate reader (Tecan GENios, Switzerland). Each experiment was performed in triplicate and repeated three times.

### *In vitro* clonogenicity assay of HepG2 cells

Clonogenicity assay was described previously^37^. Briefly, a bottom layer of 0.5% soft agar containing enriched medium (DMEM with 10% FBS) was topped with a layer of 0.3% soft agar. 1 × 10^3^ cells were plated on the top layer and cultured in a CO_2_ incubator for 2–3 weeks with humidity. Colonies were counted with naked eyes.

### Immunocytochemical or immunohistochemical staining

Immunocytochemical staining procedures were described previously^38^. Briefly, 1 × 10^5^ HepG2 cells were seeded on a glass slide overnight and rinsed once with PBS. −20 °C methanol was added immediately for fixation and washed with PBS. Detection of target proteins was performed according to the manufacturer’s instructions. Primary specific Anti-p-STAT3 antibody was purchased from Santa Cruz, CA, USA and specific antibodies to Ki67, PCNA, PSA, IL-6, IL-6R, gp130, EGFR and TGFβ were gifts from Maixin biotech., Fuzhou, China.

Paraffin sections were stained by using standard immunohistochemical procedures described previously. Briefly, mouse tissue samples were fixed with 10% formalin for 24 hrs and paraffin embedded. Four mm thick serial sections were mounted on glass slides and were immunostained with various antibodies described in the previous section following standard procedures^39^.

### Animals and tumor induction

Male BalB/c mice (21 days of age) were purchased from Slac laboratory animal, China, and were housed in a standard vivarium with 12-h light/dark cycles and free access to food and water. Animals were treated in accordance with the Guide for the Care and Use of Laboratory Animals (Ministry of Science and Technology of China, 2006) and the study was approved by Fuzhou University Institutional Animal Care and Use Committee. The preparation of DEN-induced mouse HCC was modified according to Singh *et al.*^40^ Mice of DEN-treatment group were injected intraperitoneally (i.p.) with 200 mg/kg DEN (Sigma aldrich, USA) dissolved in normal saline. After 2 weeks of DEN administration, CCl_4_ (5 mL/kg) was given 3 times a week by gavage till the end of the experiment. Similarly, control group was given a single i.p. injection of normal saline followed by gavage of corn oil three times a week. Sgp130 was given by intravenous injection of 3 μg/kg once every 3 days during the entire experiment after DEN and the first dose of CCl_4_ were given.

### Tumor injection

The method for tumor injection followed the standard procedures described previously^41^. Briefly, HepG2 cells were re-suspended with PBS and mixed at 1:1 ratio with Matrigel (BD Biosciences, USA) on ice. The cell mixture was injected subcutaneously in the bilateral flanks of male NOD/SCID mice. Three μg/kg of sgp130 was injected via tail veins into each mouse in treatment group once every three days. Mice injected with normal saline served as controls. At least six mice were included in each treatment group. Tumor size was measured with a caliper when visible nodules could be observed.

### Western blot analysis

The method of Western blot analysis was described previously^42^. Briefly, cell or tissue lysates were dissolved in PBS and separated on a 12% SDS-PAGE gel by electrophoresis. Proteins were transferred from the gel onto a nitrocellulose membrane in a semi-dry electrotransfer system. Detection of p-STAT3 was performed by immunostaining with p-STAT3 specific antibody at 1:500 dilution (Santa Cruz, CA, USA) and horseradish peroxidase-conjugated anti-IgG antibody (Maixin biotech., China). The protein bands were visualized by enhanced chemiluminesence (ECL) reagents (Amersham Pharmacia Biotech, USA).

### Determination of mRNA Expression

Total RNA was isolated from cells or liver tissue using the TRIzol^®^ Reagent kit (Invitrogen, CA, USA) according to the manufacturer’s protocol. cDNAs were synthesized from 0.5 μg of total RNA using a First Strand cDNA Synthesis Kit (MBI Fermentas, USA). mRNA levels of various genes were determined by quantitative PCR performed with SYBR Green I and ABI 7300 instrument (Manufacturer). Primers were synthesized by DNA Synthesis Center (Sangon Biotech, Shanghai, China). The sequences of primers are listed in Table 1. All mRNA signals were normalized to β-actin for comparison.

### Statistical Analysis

Data were analyzed using SPSS software (SPSS, Inc., Chicago, IL) as follows: (1) two-tailed Student t test with P < 0.05 considered statistically significant for *in vitro* cell line experiments, including qRT-PCR, cell growth assay, cell migration assay, and tube formation assay; (2) significance of associations between gene expression values was judged by a test statistic based on Pearson’s product-moment correlation coefficient.

## Additional Information

**How to cite this article**: Hong, J. *et al.* Recombinant soluble gp130 protein reduces DEN-induced primary hepatocellular carcinoma in mice. *Sci. Rep.* **6**, 24397; doi: 10.1038/srep24397 (2016).

## Figures and Tables

**Table 1:** the sequence of primers.

	forward	reverse
IL-6	5′-ATGAACTCCTTCTCCACAAGCGC-3′	5′-GAAGAGCCCTCAGGCTGGACTG-3′
IL-6R	5′-CATTGCCATTGTI%TGAGGlTC-3′	5′-AGTAGTCTGTATTGCTGATGTC-3′
gp130	5′-ACAGATGAAGGTGGGAAGGAT-3′	5′-AGATGACATGCATGAAGACCC-3′
IL-1β	5′-ACAGATGAAGTGCTCCTTCCA	5′-GTCGGAGATTCGTAGCTGGAT-3′
TNFα	5′-CCCAGGGACCTCTCTCTAATC-3′	5′-ATGGGCTACAGGCTTGTCACT-3′
Cox-2	5′-CCCTTGGGTGTCAAAGGTAA-3′	5′-GCCCTCGCTTATGATCTGTC-3′
IFNγ	5′-CTAATTATTCGGTAACTGACTTGA-3′	5′-ACAGTTCAGCCATCACTTGGA-3′
IL-10	5′CATCGATTTCTTCCCTGTGAA-3′	5′-TCTTGGAGCTTATTAAAGGCATTC-3′
IL-11	5′-TCTCTCCTGGCGGACACG-3′	5′-AATCCAGGTTGTGGTCCCC-3′
